# NOTCH1 inhibition *in vivo *results in mammary tumor regression and reduced mammary tumorsphere-forming activity *in vitro*

**DOI:** 10.1186/bcr3321

**Published:** 2012-09-19

**Authors:** Matthew J Simmons, Ryan Serra, Nicole Hermance, Michelle A Kelliher

**Affiliations:** 1 Department of Cancer Biology, University of Massachusetts Medical School, 364 Plantation Street, Worcester, MA 01605 USA; 2 Program in Gene Function and Expression, University of Massachusetts Medical School, 364 Plantation Street, Worcester, MA 01605 USA

## Abstract

**Introduction:**

NOTCH activation has been recently implicated in human breast cancers, associated with a poor prognosis, and tumor-initiating cells are hypothesized to mediate resistance to treatment and disease relapse. To address the role of NOTCH1 in mammary gland development, transformation, and mammary tumor-initiating cell activity, we developed a doxycycline-regulated mouse model of NOTCH1-mediated mammary transformation.

**Methods:**

Mammary gland development was analyzed by using whole-mount analysis and by flow cytometry in nulliparous transgenic mice maintained in the presence/absence of doxycycline (or intracellular NOTCH1). Mammary tumors were examined histologically and immunophenotyped by staining with antibodies followed by flow cytometry. Tumors were transplanted into mammary fat pads under limiting dilution conditions, and tumor-initiating cell frequency was calculated. Mammary tumor cells were also plated *in vitro *in a tumorsphere assay in the presence/absence of doxycycline. RNA was isolated from mammary tumor cell lines cultured in the presence/absence of doxycycline and used for gene-expression profiling with Affymetrix mouse arrays. NOTCH1-regulated genes were identified and validated by using quantitative real-time polymerase chain reaction (PCR). Mammary tumor-bearing mice were treated with doxycycline to suppress NOTCH1 expression, and disease recurrence was monitored.

**Results:**

Similar to published studies, we show that constitutive expression of human intracellular NOTCH1 in the developing mouse mammary gland inhibits side branching and promotes luminal cell fate. These mice develop mammary adenocarcinomas that express cytokeratin (CK) 8/18. *In vivo *limiting-dilution analyses revealed that these mammary tumors exhibit functional heterogeneity and harbor a rare (1/2,978) mammary tumor-initiating cell population. With this dox-regulated NOTCH1 mammary tumor model, we demonstrate that NOTCH1 inhibition results in mammary tumor regression *in vivo *and prevents disease recurrence in four of six tumors tested. Consistent with the *in vivo *data, NOTCH1 inhibition reduces mammary tumorsphere activity *in vitro*. We also identify the embryonic stem cell transcription factor *Nanog *as a novel NOTCH1-regulated gene in tumorspheres and in mouse and human breast cancer cell lines.

**Conclusions:**

These data indicate that NOTCH1 inhibition results in mammary tumor regression *in vivo *and interferes with disease recurrence. We demonstrate that NOTCH1-transformed mouse mammary tumors harbor a rare mammary tumor-initiating population and that NOTCH1 contributes to mammary tumor-initiating activity. This work raises the possibility that NOTCH therapeutics may target mammary tumor-initiating cells in certain human breast cancer subtypes.

## Introduction

NOTCH activation has been implicated in several malignancies; notably T-cell acute lymphoblastic leukemia, chronic lymphocytic leukemia, glioblastoma, and breast cancer [1-11]. Overexpression of NOTCH receptors has been implicated in ductal carcinoma *in situ *(DCIS) and invasive breast cancer [12,13], and high levels of the NOTCH ligand JAG1 appear to predict a poor overall survival [14]. High NOTCH1-receptor levels have been linked with basal-like, triple-negative (estrogen receptor-, progesterone receptor-, and HER2-negative) breast cancer, and NOTCH1 levels correlate with abbreviated survival [15]. More recently, silencing of Lunatic Fringe, the glycosylase that regulates NOTCH1 ligand activity, has been observed in patients with basal-like breast cancer, and increased levels of intracellular NOTCH1 are detected in these patients' cells [16]. NOTCH1 activity levels have also been shown to correlate with the development of resistance to conventional as well as to targeted therapies [17-20], leading us to hypothesize that NOTCH1 may contribute to therapeutic resistance and disease recurrence by regulating breast tumor-initiating cell activity.

NOTCH pathway activation is triggered on ligand-receptor interaction. Mammals possess four NOTCH receptors (NOTCH 1-4) and five ligands (JAG1, 2, DELTA-like (DLL) 1, 3, and 4). Ligand binding stimulates two sequential proteolytic cleavages; the first in the extracellular domain mediated by metalloproteases of the ADAM family, and the second within the transmembrane domain mediated by the gamma-secretase complex. The second cleavage allows the release and translocation of the intracellular domain of NOTCH into the nucleus, where it associates with the CBF1/RBP-Jκ/Suppressor of Hairless/LAG-1 (CSL) repressor and on the recruitment of co-activators Mastermind-like 1 (MAML1) and CBP/p300 induces expression of NOTCH target genes, including HES1, HEY2, DELTEX1, and c-MYC [21]. Gamma secretase inhibitors (GSIs) have been shown to inhibit Notch1 and to have antileukemia activity *in vivo *[22-24].

Constitutive Notch1 signaling in the normal mouse mammary stem cell (MaSC) has been shown to stimulate differentiation toward a luminal fate, whereas suppression of Notch signaling in MaSC via *CSL *knockdown results in the expansion of the MaSC compartment [25,26]. These studies implicate Notch1 pathway activation in mouse luminal progenitor expansion and differentiation. NOTCH pathway activation has also been shown to enhance human mammosphere formation, which likely reflects NOTCH pathway effects on the human mammary stem or progenitor cells [27].

In addition to the Notch receptor family, the gamma-secretase complex regulates the expression of ErbB4, CD44, and E cadherin, cell-surface receptors known to contribute to tumor growth, migration, and invasion [28-30]. Thus, experiments that use GSIs to determine the effect(s) of Notch inhibition on tumor growth likely affect the stability of other substrates relevant to mammary gland transformation. Moreover, GSI studies fail to reveal which Notch receptor family member mediates the effects on tumor growth/survival.

To determine the specific effects of NOTCH1 activation/inhibition on bulk mammary tumor growth and on mammary tumor-initiating cells, we generated a mouse mammary tumor model in which human intracellular NOTCH1 expression is doxycycline regulated. Consistent with previous reports [25,26,31-33], we demonstrated that NOTCH1 signaling stimulates luminal cell fate and results in luminal lineage transformation. *In vivo *limiting-dilution analysis reveals that only a small percentage (~1/3,000) of NOTCH1-driven mammary tumor cells are capable of transplanting disease, revealing that mammary tumor-initiating cells contribute to disease pathogenesis in this model. We also demonstrated that NOTCH1 signaling is required for mammary tumor-initiating cell activity, as NOTCH1 inhibition results in rapid mammary tumor regression and delays and, in some cases, prevents disease recurrence. By using gene-expression profiling, we identified the embryonic stem cell transcription factor *Nanog *as a novel NOTCH1-regulated gene in mammary tumor cells. These data demonstrate that NOTCH1 activation stimulate luminal lineage development and implicate NOTCH1 in the regulation of mammary tumor-initiating activity.

## Materials and methods

## Mice histopathology and immunohistochemistry

The MMTV-tTA (C57BL/6) and tet-op-Notch^IC ^(FVB/N) mice were described previously [34,35] and generously provided by D. Tenen and A.J. Capobianco, respectively. Mice were maintained in mating pairs of MMTV-tTA/TOP-ICN1 females and MMTV-tTA males, and females were monitored weekly for signs of disease. Mice were killed when total tumor volume surpassed 1,000 mm^3^, as determined by external measurement by using calipers. Tumors were fixed in 10% formalin for 4 hours at room temperature or overnight at 4°C, then transferred to 70% ethanol and maintained at 4°C until mounting in paraffin and sectioning. Sections were stained with antibodies against mouse keratin 5 (PRB-160P; Covance, Princeton, NJ), keratin 8/18 (GP11; Progen Biotechnik, Heidelberg, Germany), keratin 14 (Clone LL002; Thermo Scientific, Waltham, MA) or estrogen-receptor alpha (sc-8005; Santa Cruz Biotechnology, Santa Cruz, CA). All animal experiments were reviewed and approved by the Institutional Animal Care and Use Committee (IACUC) of the University of Massachusetts Medical School.

### Establishment of tumor-derived cell lines

Primary mouse mammary tumors were minced with a razor blade and digested in DMEM/F12 (1:1) media supplemented with 5% fetal bovine serum (FBS) and 2 mg/ml collagenase (Gibco, Grand Island, NY) for 2 hours at 37°C. Samples were spun down and washed 5 times in PBS supplemented with 5% FBS and then plated onto 10-cm collagen-coated plates (BD Biosciences, San Diego, CA) in DMEM/F12 (1:1) media supplemented with 2% FBS and penicillin/streptomycin. Cell clusters were left undisturbed for 3 days, with subsequent media changes every 3 days, gradually increasing FBS concentration to 10%. When confluent, cells were passaged at a 1:2 or 1:3 dilutions after a 5-minute incubation with Versene (Gibco, Grand Island, NY) and maintained on standard tissue-culture plates.

### Mammary fat-pad transplants

For limiting-dilution studies, primary mammary tumors were digested as previously described [36,37]. In brief, tumors were sequentially digested at 37°C in 300 U/ml collagenase (Gibco, Grand Island, NY) plus 100 U/ml hyaluronidase (Sigma, St. Louis, MO) (2 hours), 0.25% trypsin (Gibco, Grand Island, NY) (2 minutes), and 5 mg/ml Dispase II (Roche, Indianapolis, IN) plus 0.1 mg/ml DNaseI (Sigma, St. Louis, MO) (5 minutes). Cells were filtered through a 40-μm mesh, counted, and enzymatic digestion was repeated until the suspension was >95% single cells. Serial dilutions were resuspended in 35 μl gelatinous protein mixture (Matrigel; BD Biosciences, San Diego, CA) and injected into the thoracic mammary fat pads of nude mice. For tumor-derived cell-line tumorigenicity assays, cultured cells were lifted by using Versene (Gibco, Grand Island, NY), followed by trypsin-EDTA treatment and filtering through a 40-μm mesh. Approximately 10^6 ^cells were resuspended in 35 μl Matrigel and injected into the thoracic mammary fat pads of nude mice.

### Tumorsphere cultures

Single-cell suspensions (>95% single cells) were generated from primary mammary tumors as described earlier. Cells were then plated in 3 ml defined tumorsphere media [38] plus 0.5% methylcellulose (R&D Systems, Minneapolis, MN) per well of a six-well ultra-low-attachment plate (Fisher Scientific, Pittsburgh, PA), at a concentration of 20,000 cells/ml. For doxycycline-treated samples, 2 μg/ml doxycycline was added to the culture media at the time of plating.

### Flow cytometry

Tumor samples, thoracic and inguinal mammary fat pads from nulliparous females, or tumor-derived cell lines were subjected to enzymatic digestion to create a single-cell suspension as described earlier. Antibodies against mouse antigens were purchased from BD Pharmingen (San Diego, CA) unless otherwise noted, and included Ter-119-PE, CD31-PE, CD45R-PE, CD61-FITC, CD24-biotin, streptavidin-APC, and CD29-PE-Cy7 (eBioscience, San Diego, CA). Cells were stained in PBS at 4°C for 25 minutes and analyzed live. For cell-cycle analysis, cells were fixed in 70% ethanol, stained with propidium iodide (PI), and analyzed for DNA content.

### Microarray analysis

Tumor-derived cell lines 8534 and 8542 were left untreated or treated with 2 μg/ml doxycycline for 24 hours. Cells were collected by scraping, and total RNA was isolated by using Trizol (Invitrogen, Grand Island, NY). After real-time PCR validation of NOTCH1 target-gene modulation, RNA samples were further purified by using the RNAeasy Mini kit (Qiagen, Valencia, CA) and hybridized to Affymetrix mouse genome 430A2.0 arrays (Affymetrix, Santa Clara, CA, USA). Raw data were processed with MAS5 analysis, and genes showing a >2.0-fold change in both cell lines were considered targets of interest. The data from these arrays have been deposited in the NCBI Gene Expression Omnibus and are accessible through GEO Series accession number GSE34146.

### Quantitative RT-PCR

Total RNA from cells was extracted by using Trizol (Invitrogen, Grand Island, NY). cDNA was prepared with the Superscript First Strand Synthesis kit (Invitrogen, Grand Island, NY), and PCR was carried out with SYBR Green (Qiagen, Valencia, CA). The following primers were used in this study: *hey1*, 5 -TGAGCTGAGAAGGCTGGTAC-3 (Forward) and 5 -ACCCCAAACTCCGATAGTCC-3 (Reverse); *deltex1*, 5 -TGCCTGGTGGCCATGTACT (Forward) and 5 -GACACTGCAGGCTGCCATC-3 (Reverse); β*-actin*, 5 -CGAGGCCCAGAGCAAGAGAG-3 (Forward) and 5 -CGGTTGGCCTTAGGGTTCAG-3 (Reverse); *c-myc*, 5 -CTGTTTGAAGGCTGGATTTCCT-3 (Forward) and 5 -GTCGTGGCTGTCTGCGG-3 (Reverse); *hes1*, 5 -AAGACGGCCTCTGAGCACA-3 (Forward) and 5 -CCTTCGCCTCTTCTCCATGAT-3 (Reverse); *nanog*, 5 -TCTTCCTGGTCCCCACAGTTT-3 (Forward) and 5 -GCAAGAATAGTTCTCGGGATGAA-3 (Reverse). The *nanog *primer set (PrimerBank ID 31338864a1) was obtained from PrimerBank [39].

### Western blotting

Protein was isolated from cells collected by using Versene, washed in PBS, and lysed in radioimmunoprecipitation assay (RIPA) buffer containing protease inhibitor tablets (Roche, Indianapolis, IN). Fifteen to twenty-five micrograms of total protein was resolved via 9% sodium dodecylsulfate-polyacrylamide gel electrophoresis (SDS-PAGE), as previously described [40]. Blots were probed with antibodies against intracellular NOTCH1 (provided by Jon Aster), active NOTCH1 (2421; Cell Signaling Technology, Danvers, MA), Nanog (AB9220; Millipore, Billerica, MA), cytokeratin 8/18 (GP11; Progen Biotechnik, Heidelberg, Germany), caspase-3 (Cell Signaling, Danvers, MA), and α-tubulin (T5168; Sigma, St. Louis, MO) or Erk1/2 (Cell Signaling, Danvers, MA) to control for equal loading. For MDA-MB-231 study, cells were left untreated or treated with the gamma-secretase inhibitor (GSI) Compound E (Axxora, Farmingdale, NY) at 10 μ*M *for the times noted, before cells were collected, and analyzed as described earlier.

### Mammary fat pad whole mounting

Inguinal fat pads were isolated from mice administered doxycycline-treated sugar water (10 μg/ml) for various time periods, spread on glass slides, and fixed in glacial acetic acid/ethanol (1:3) overnight. Samples were washed in 70% ethanol for 15 minutes, rinsed in distilled water for 5 minutes, and stained overnight in carmine alum solution. Samples were then dehydrated and transferred to xylene overnight for delipidation. Whole mounts were briefly air dried, and coverslips were mounted by using Permount (Fisher Scientific, Pittsburgh, PA).

### MTT assay

MTT cell-viability assays were performed as previously described [22], with the following modifications. In brief, approximately 10^4 ^cells/200 μl of a cell suspension were plated in a 96-well flat-bottom plate, ~16 hours before treatment. Cells were then left untreated or treated with 2 μg/ml doxycycline. After 72 hours, 20 μl of a 5-mg/ml 3-(4,5-dimethylthiazol-2-y1)-2,5-diphenyl tetrazolium bromide (MTT) solution (Sigma-Aldrich, St. Louis, MO) was added and incubated for 4 hours at 37°C. Media was then removed, and the reagent was solubilized with 100 μl dimethyl sulfoxide (DMSO) (Sigma-Aldrich, St. Louis, MO) and incubated for 10 minutes at room temperature. Plates were then analyzed at A595 wavelength. Data are plotted as absorbance and are the average of five independent experiments. The *P *values were calculated by using a two-tailed distribution and paired Student *t *test.

### Tumorsphere immunofluorescence

For detection of Keratin 8/18 and Nanog protein expression, primary mammary tumor cells were grown under mammosphere culture conditions for 7 days. Spheres were collected by pipette under a dissection microscope, pooled, washed, and digested in 0.25% trypsin (Gibco, Grand Island, NY) at 37°C for 5 minutes, and the resulting cell suspension was cytospun onto coverslips. In parallel, tumor-derived cell line 8542 was plated onto glass coverslips and allowed to grow for 48 hours before processing. Cell line and primary samples were fixed in 4% paraformaldehyde and permeabilized for 10 minutes in phosphate-buffered saline (PBS) containing 0.2% Triton X-100. Cells were washed with PBS containing 0.02% Triton X-100 and 10% FBS, followed by incubation with α-Keratin 8/18 (Progen Biotechnik, Heidelberg, Germany) and α-Nanog (Millipore, Billerica, MA) antibodies or IgG controls (Santa Cruz, Santa Cruz, CA) for 1 hour at room temperature. Cells were stained with FITC- or rhodamine-conjugated secondary antibodies (Santa Cruz, Santa Cruz, CA), and coverslips were mounted with Permount (Fischer Scientific, Pittsburgh, PA) and photographed under ultraviolet illumination at a magnification of 600X.

For detection of cell-surface marker CD61, tumorspheres were grown, collected, and digested with trypsin as described earlier to break up the spheres into loose aggregates of cells. Cells were resuspended in PBS plus 5% FBS and stained with CD61-FITC (1:75, eBioscience, San Diego, CA) or IgG-FITC at 4°C for 1 hour in the dark, and washed. Cells were then cytospun onto glass coverslips and mounted with DAPI-containing mounting medium and photographed under ultraviolet illumination at a magnification of 600X.

## Results

### NOTCH1 expression impairs ductal side branching and expands the mature luminal population

**Figure 1:**
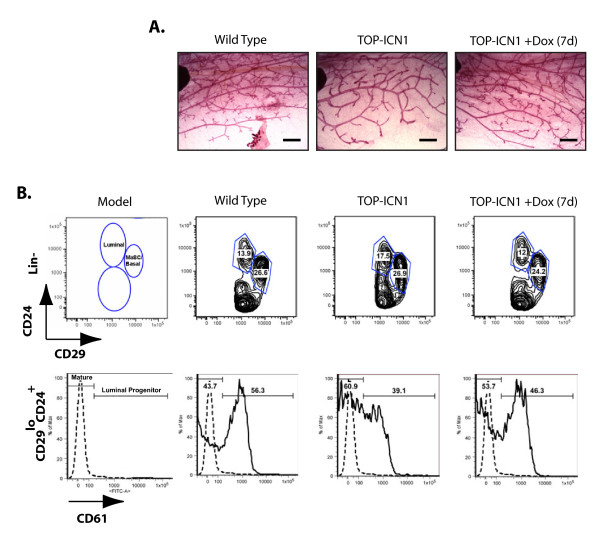
**Notch1 expression impairs ductal side branching and stimulates luminal differentiation**. **(A) **NOTCH1 inhibits mammary gland branching morphogenesis. Representative (*n *= 3) whole-mount analysis of mammary fat pads from wild-type, premalignant 3-month-old MMTV-tTA/Tet-Op-NOTCH1 or MMTV-tTA/Tet-Op-NOTCH1 mice treated with doxycycline for 7 days. Scale bars, 1,000 μm. **(B) **Constitutive NOTCH1 signaling promotes luminal differentiation. Matching fluorescence-activated cell sorting (FACS) histograms of mammary epithelial cells derived from the mice described in A. Cells were stained with antibodies against Ter119, CD45, and CD31, and the lineage-negative cells were then stained with CD29 and CD24 (top), and CD61 antibodies (bottom) and analyzed with flow cytometry. Dashed line, staining with an isotype-matched control.

To investigate the role of NOTCH1 in mammary gland development and tumor progression, we mated mice expressing the human intracellular form of NOTCH1 under control of a tet-responsive promoter (Tet-Op-ICN1) with mice expressing the tet Transactivator under control of the MMTV promoter (MMTV-tTA) [34,41], allowing us to modulate expression of intracellular NOTCH1 in the developing mouse mammary gland. In the absence of doxycycline, these mice express intracellular NOTCH1, whereas addition of doxycycline to the drinking water suppresses NOTCH1 expression. Consistent with similar models [31-33], MMTV-tTA/TOP-ICN1 female mice appear normal at birth, and transgenic females had fully functional mammary glands capable of nursing their young. Whole-mount analysis of the mammary fat pad of 8- to 12-week-old nulliparous females revealed decreased ductal side branching in developing transgenic mammary fat pads compared with that in wild-type littermate controls (Figure 1A). Doxycycline administration suppressed intracellular NOTCH1 expression (see Additional file 1) and reversed this phenotype, with dox-treated mice exhibiting decreased side branching compared with untreated transgenic littermates (Figure 1A).

Notch signaling is critical for proper mammary lineage specification and differentiation. Suppression of Notch signaling in the mammary epithelium of *Rbp-Jκ^fl/fl^;MMTV-Cre *mice, or knockdown of *Cbf-1 *in sorted mammary stem cells (MaSCs), leads to a block in mammary differentiation, resulting in the expansion of the MaSC population [25,26]. Conversely, constitutive Notch1 activation promotes luminal cell commitment, at the expense of the myoepithelial population, and confers self-renewal capacity on luminal progenitor cells in Matrigel assays [25].

To examine further the effects of NOTCH1 activity on mammary developmental fate and transformation, we stained cells isolated from the developing mammary gland with antibodies to surface markers and analyzed with flow cytometry. Published studies have shown that expression of CD29 (β_1_-integrin) and CD24 (heat-stable antigen) can be used to distinguish the luminal cell population (lin^-^CD29^lo^CD24^+^) from the combined mammary stem cell and basal cell subpopulations (lin^-^CD29^hi^CD24^+^). Analysis of the mammary fat pad of the premalignant MMTV-tTA/TOP-ICN1 mouse revealed a slightly expanded luminal cell population compared with wild-type littermates (average 22.5% versus 15.7%; SEM, 2.29% versus 1.09%; *n *= 5) (Figure 1B, top panels). Further separation of the luminal population into luminal progenitors and mature luminal cells on the basis of CD61 expression levels [42] reveals an average 47% (SEM 10.4%, *n *= 5; *P* < 0.05) decrease in the proportion of CD61^+ ^cells in mice expressing intracellular NOTCH1 (Figure 1B, bottom panels). Treatment of a premalignant MMTV-tTA/TOP-ICN1 littermate with doxycycline for 7 days before analysis results in a decrease in the relative proportion of luminal cells to levels comparable to those observed in control littermates. A shift in CD61 expression is also observed in the dox-treated mice, revealing a decrease in mature luminal cells and a corresponding increase in luminal progenitors (Figure 1B, top and bottom panels). The decrease in the frequency of CD61^+ ^luminal progenitors in the NOTCH1 transgenic mice was not due to NOTCH1 effects on CD61 mRNA levels (see Additional file 2).

The change in the percentage of luminal progenitors was highly reproducible in the five experiments performed, and the data were statistically significant (*P* < 0.05). The decreased frequency resulted in modest decreases in the absolute number of luminal progenitors in the NOTCH1 transgenic mammary gland (37,167 ± 6,406 SEM compared with wt control, 43,159 ± 5,643 SEM; *n *= 3 mice of each genotype); however, this decrease in luminal progenitor numbers was not manifest in statistically significant differences in colony-forming activity *in vitro *(data not shown). Collectively these data are consistent with other reports [25,26] that suggest that Notch1 promotes a luminal cell fate, and that the majority of these cells are fully differentiated in the premalignant gland.

### NOTCH1 expression results in mammary gland transformation

**Figure 2:**
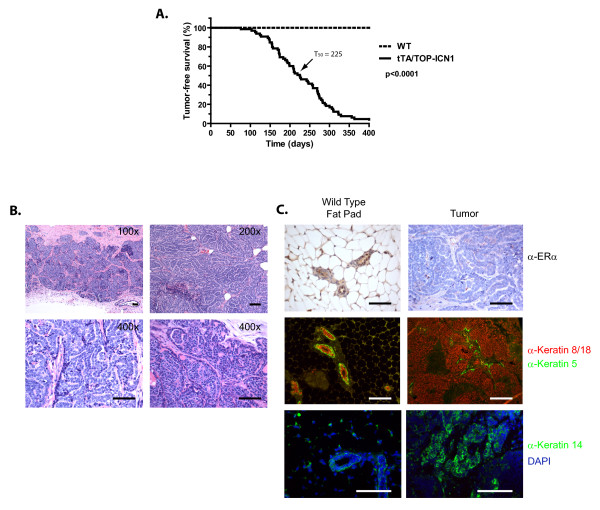
**Deregulated NOTCH1 expression results in mammary gland transformation**. **(A) **Constitutive NOTCH1 signaling results in mammary tumor formation. A cohort of wild-type (*n *= 15) and MMTV-tTA/TOP-ICN1 (*n *= 65) mice were maintained under mating conditions and monitored for tumor formation. The proportion of tumor-free mice was plotted by using the Kaplan-Meier software. **(B) **NOTCH1 expression leads to tubular adenocarcinomas. Sections from MMTV-tTA/TOP-ICN1 primary mammary tumors were fixed in 10% formalin, paraffin embedded, sectioned, and stained with hematoxylin and eosin (H&E). Analysis of tumors showed multiple initiations within a single fat pad, with cordlike structures of two or three cell layers separated by stroma. Scale bars, 100 μm. **(C) **NOTCH1-transformed mammary tumors are ERα-negative and express the luminal lineage cytokeratins 8 and 18. Tumor sections from wild-type mammary fat pad (left) or primary tumor (right) were stained with antibodies against ERα (top), Cytokeratin 8/18 and Cytokeratin 5 (middle), or Cytokeratin 14, and counterstained with DAPI (bottom). Scale bars, 100 μm.

Previous Notch-driven mammary-tumor mouse models display varying phenotypes, ranging from mammary hyperplasia and DCIS in nulliparous mice to lactation-dependent regressing tumors and nonregressing invasive adenocarinomas [8,31-33]. To identify the target cell of NOTCH1-mediated transformation, we monitored a cohort of MMTV-tTA/TOP-ICN1 transgenic females and their wild-type littermates for disease development. Consistent with the MMTV expression in T cells and the demonstrated role of *Notch1 *as a T-ALL oncogene [4,5,40], 18% of the transgenic mice developed T-ALL-like disease and were excluded from this study (data not shown). The remaining cohort maintained under mating conditions developed mammary adenocarcinomas by 12 months of age, with a median tumor-free survival of 225 days and a penetrance of >90% (Figure 2A). Nulliparous females also developed mammary tumors with reduced penetrance and after an average latency of 353 days (see Additional file 3). Some mice displayed tumors in multiple mammary glands, although the majority (59%) of mice had a single mammary tumor.

Multiple, focal tumor initiations were observed within a single fat pad, composed of intertwining cords of neoplastic cells forming tubules two or three cells wide (Figure 2B). These strands are further delineated by a fibrovascular stroma, and some strands appear to contain a third cell population resembling myoepithelium. Some tumors also exhibited microcalcification and infiltrated the adjacent skeletal muscle (data not shown). The tumor cells displayed large nuclei with sparse cytoplasm and had a low mitotic rate.

Recent studies suggest that deregulated NOTCH1 signaling may contribute to relapse and/or chemotherapeutic resistance in triple-negative basal-like breast cancers, and that NOTCH signaling may be required to maintain ER-negative tumors [15,18,20]. Consistent with these findings and other mouse mammary tumor models, immunohistochemical analysis failed to detect ERα expression in these NOTCH1-induced mouse mammary tumors, whereas ERα reactivity was observed in the luminal cells of the wild-type mammary fat pad, as expected (Figure 2C, top panels). Keratin 14 (K14) is expressed in basal cells in the mouse mammary epithelium [43], and importantly, K14 is expressed by human and mouse mammary stem cell populations [44-46]. Immunohistochemical analysis of tumor sections with antibodies against Cytokeratins K8/18, K5, and K14 revealed that the NOTCH1-induced mammary tumors consist of K8/18-expressing cells, confirming that the tumor consists of primarily luminal epithelial cells (Figure 2C, middle right panel). Many tumors also contained rare cells (<1%) that stained positive for K14 or K5 (Figure 2C, right middle and bottom panel).

### NOTCH1 inhibition induces apoptosis of mouse mammary tumor cell lines

**Figure 3:**
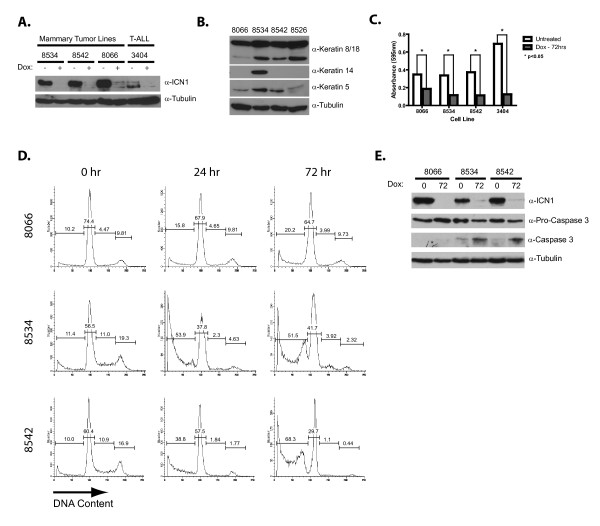
**NOTCH1 inhibition induces apoptosis of mammary tumor cells**. **(A) **NOTCH1 expression is rapidly suppressed in doxycycline-treated mammary tumor cell lines. Cell lines were left untreated or treated with doxycycline (2 μg/ml) for 24 hours, and lysates were analyzed for ICN1 expression with immunoblotting. A doxycycline-regulated T-ALL cell line 3404 is included as a control. **(B) **Mammary tumor cell lines express cytokeratin 8/18, but have variable expression of basal cytokeratins 5 and 14. Cell-line lysates were analyzed for CK8/18, CK5, and CK14 expression with immunoblotting. **(C) **NOTCH1 inhibition arrests mammary tumor cell growth. Three mammary tumor cell lines (8066, 8534, and 8542) and T-ALL cell line 3404 were left untreated or treated with doxycycline (2 μg/ml) for 72 hours, and cell growth was measured by using an MTT assay. The figure represents the average of three independent experiments, statistically analyzed by the Student paired t test. **(D) **NOTCH1 inhibition induces apoptosis of multiple mammary tumor cell lines. Tumor-derived cell lines 8066, 8534, and 8542 were treated with 2 μg/ml doxycycline or left untreated for 0, 24, or 72 hours. Cells were assayed for DNA content by staining with PI followed by flow cytometry. **(E) **NOTCH1 inhibition results in caspase 3 activation. Cells were left untreated or treated with doxycycline (2 μg/ml) for 72 hours, and lysates were analyzed for cleaved or total caspase-3 levels with immunoblotting.

Primary mouse and human T-ALL cells are sensitive to the effects of Notch1 inhibition and undergo G_1 _arrest and/or apoptosis [4,5]. To determine whether mammary tumor growth/survival remains NOTCH1 dependent, we established cell lines from primary MMTV-tTA/TOP-ICN1 mammary tumors. Each cell line retained doxycycline responsiveness, as treatment with dox for 24 hours resulted in decreased ICN1 expression compared with untreated controls (Figure 3A). Consistent with tumor immunohistochemistry data, all four mammary tumor-derived cell lines expressed cytokeratin 8/18 (Figure 3B). Interestingly, the mammary tumor cell lines also expressed the basal markers K14 and/or K5 (Figure 3B), indicating that conversion to culture may select for a rare, double-positive mammary tumor cell. Transplantation of these mammary tumor cell lines into nude mice resulted in growth of mammary tumors that appeared histologically identical to primary tumors (see Additional file 4).

To determine the consequences of NOTCH1 inhibition on mammary tumor growth/survival, we treated the mammary tumor cell lines with doxycycline for 72 hours and performed an MTT analysis. Doxycycline treatment resulted in >50% (*P* < 0.05) decrease in mammary tumor cell viability (Figure 3C). Cell-cycle analysis of the doxycycline-treated mammary tumor cell lines revealed increases in the sub-G_1 _population, with little to no evidence of cell-cycle arrest (Figure 3D). Consistent with these results, cleaved (active) caspase 3 was detected in two of three doxycycline-treated mammary tumor cell lines examined (Figure 3E). These studies reveal that a sustained NOTCH1 signal is required for the maintenance of the mammary tumor cell lines.

### NOTCH1 activation in mammary tumor cells induces Nanog expression

**Figure 4:**
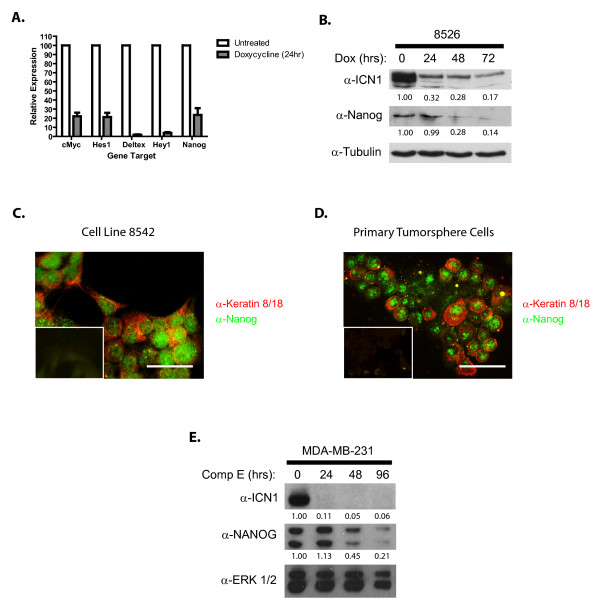
**NOTCH1 regulates Nanog expression in mouse mammary tumor cells**. **(A) **Nanog expression is suppressed on NOTCH1 inhibition. RNA was harvested from tumor cell lines left untreated or treated with doxycycline (2 μg/ml) for 24 hours and *c-Myc, Hes1, Deltex1, Hey1*, and *Nanog *expression levels determined with quantitative real-time PCR. Expression is normalized to the untreated control. **(B) **Doxycycline treatment reduces Nanog protein levels. The mouse mammary tumor lines (8542, 8526) were left untreated or treated with doxycycline (2 μg/ml) for the time periods indicated. Lysates were analyzed for ICN1 and Nanog expression with immunoblotting, and relative band intensities were quantified. **(C**, **D) **Nanog is expressed in the nuclei of NOTCH1-transformed mouse mammary tumor cell lines and primary tumorspheres. The mouse mammary tumor cell line 8542 was grown on coverslips, and primary tumorspheres were dissociated and centrifuged onto slides, followed by fixation in 4% paraformaldehyde. The cells were then permeabilized with Triton X-100 and immunostained by using antibodies against cytokeratin 8/18 (red) or Nanog (green). Cells were photographed at 400 × magnification. Scale bars, 100 μm. **(E) **NOTCH1 regulates NANOG expression in human breast cancer cells. The human basal-like breast cancer cell line MDA-MB-231 was left untreated or treated with the γ-secretase inhibitor Compound E (10 μ*M*) for the time periods indicated, and intracellular NOTCH1 and NANOG expression levels examined with immunoblotting.

Previous studies have demonstrated the importance of *c-Myc *as a Notch1-regulated gene in T-ALL [40,47,48] and in mouse mammary tumorigenesis [32]. To identify novel NOTCH1-regulated genes important in mouse mammary tumorigenesis, we performed a microarray analysis on untreated and doxycycline-treated mammary tumor cell lines (GEO accession number GSE34146). In addition to decreased expression of known NOTCH1-regulated genes, such as *Hes1, Deltex1, Hey1*, and *c-Myc*, suppression of NOTCH1 signaling resulted in a ninefold (*P* < 0.005) decrease in the expression of Nanog, a transcription factor required for the maintenance of embryonic stem (ES) cell pluripotency [49-51]. We validated this finding in multiple NOTCH1-transformed mammary tumor cell lines using quantitative real-time PCR and observed on average a fivefold decrease in *Nanog *mRNA levels in doxycycline-treated mammary tumor cells (Figure 4A). Decreased Nanog protein levels were also observed in doxycycline-treated mammary tumor cultures and correlated with decreased ICN1 expression (Figure 4B). We were, however, unable to detect Nanog expression in primary NOTCH1-transformed mammary tumors by using immunohistochemistry or quantitative real-time PCR (not shown). We hypothesize that Nanog expression may be limited to a subset of tumor cells and therefore plated primary tumors under tumorsphere conditions to select for mammary tumor-initiating cells. Nanog expression was detected in the nuclei of NOTCH1-transformed mammary tumor cell lines and in primary tumorspheres (Figure 4C, D).

To determine whether NOTCH1 contributes to the regulation of NANOG in human breast cancer cells, we treated the basal-like human breast cancer cell line MDA-MB-231 with the γ-secretase inhibitor (GSI) Compound E to interfere with NOTCH1 processing and assayed NANOG expression levels. We observed a 10-fold decrease in NANOG protein levels in the GSI-treated cells (Figure 4E), suggesting that NANOG may be NOTCH1-regulated in mouse and human breast cancer cells. Although conserved CSL sites were found in the mouse and human NANOG regulatory regions, we were unable to detect intracellular NOTCH1 binding to the mouse *Nanog *regulatory region, suggesting that NOTCH1 may indirectly regulate *Nanog *expression (data not shown).

### NOTCH1-induced mammary tumors consist of a mix of luminal progenitors and mature luminal cells

**Figure 5:**
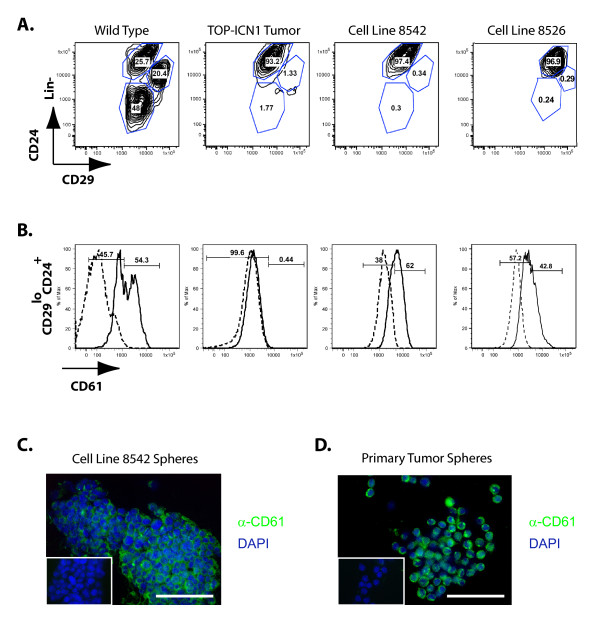
**NOTCH1-transformed mammary tumors consist of mature luminal and luminal progenitor cells**. **(A) **MMVT-tTA/TOP-ICN1 tumors and tumor cell lines are derived from luminal cells. Representative FACS profiles (*n *= 3) showing the expression of CD29 and CD24 in the CD45^-^CD31^-^Ter119^- ^(Lin^-^) population of the wild-type mammary gland, NOTCH1-transformed mammary tumor, or mammary tumor-derived cell line 8542. **(B) **Primary tumors consist of mature luminal cells, whereas tumor-derived cell lines consist of luminal progenitors. Representative histogram showing the CD61 expression levels within the Lin^-^CD29^lo^CD24^+ ^luminal cell populations shown in (A). *Dashed line*, staining observed with isotype-matched negative control. **(C**, **D) **Tumorsphere culture of primary NOTCH1-transformed mammary tumors enriches for CD61^+ ^cells. Tumor-derived cell lines (C) or primary mammary tumor cells (D) or were grown under tumorsphere conditions for 7 days, centrifuged onto slides, and stained with an isotype-matched control or anti-CD61-FITC antibody. Representative fields are shown at 400 × magnification (isotype control is shown in inset). Scale bars, 100 μm.

Mammary tumors derived from MMTV-*Wnt-1*, MMTV-*Neu*, and *p53^+/- ^*mice exhibit uniform Lin^-^CD29^lo^CD24^+ ^cell-surface marker profiles, but differ in CD61 cell-surface expression levels [52]. These findings, in conjunction with the differences seen between developing mammary glands in NOTCH1 transgenic mice and wild-type littermates (Figure 1), led us to hypothesize that NOTCH1-induced mammary tumors might express a distinct luminal surface-marker profile. Analysis of CD24 and CD29 surface-marker expression on the lineage-negative population of NOTCH1 mammary tumor cells revealed expression of a luminal cell profile (Lin^-^CD24^+^CD29^lo^), compared with wild-type mammary cells (Figure 5A and 5B). When the Lin^-^CD24^+^CD29^lo ^population is further analyzed for CD61 expression, we find that these mammary tumors do not appear to express CD61 (Figure 5B). Interestingly, when we analyzed tumor-derived cell lines 8542 and 8526 with flow cytometry, we found that the two cell lines are composed almost exclusively of luminal cells, but that unlike the primary tumor, the cell lines consist predominantly of CD61-positive cells (Figure 5B, C). Based on these data, we hypothesized that CD61^+ ^cells are present at low frequency in the primary mammary tumors. Consistent with this hypothesis, CD61^+ ^cells can be readily detected when primary mammary tumors are cultured under tumorsphere conditions (Figure 5D). These data indicate that the NOTCH1-induced mammary tumors are composed of a mixed population of luminal progenitors and mature luminal cells, and that conversion to culture selects for the luminal progenitors.

### NOTCH1 inhibition results in mammary tumor regression and delays disease recurrence

**Figure 6:**
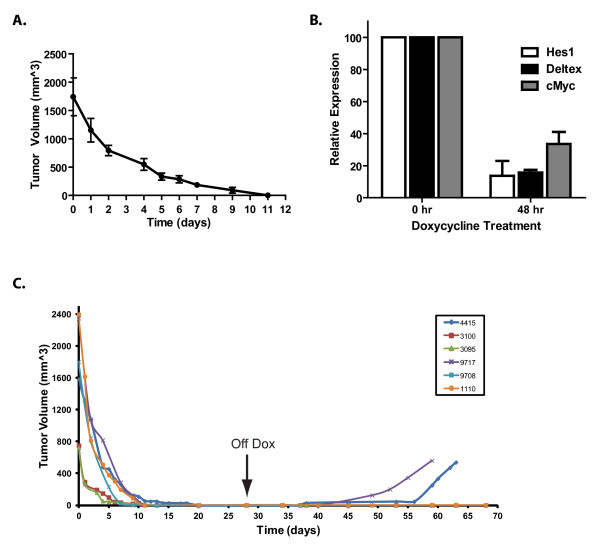
**NOTCH1 inhibition results in mammary tumor regression *in vivo *and reduces tumorsphere activity *in vitro***. **(A) **NOTCH1 inhibition *in vivo *results in rapid mammary tumor regression. Tumor-bearing mice (*n *= 5) were treated with doxycycline (10 μg/ml) for 11 days, and tumor volume was monitored externally by using calipers. The graph shows average tumor volume over time and standard error. **(B) **Doxycycline treatment results in decreased NOTCH1 target-gene expression. Total RNA was harvested from mammary tumors isolated from mice left untreated or treated with doxycycline (10 μg/ml) for 24 or 48 hours. *Hes1, Deltex1*, and *c-Myc *expression levels were quantified by using real-time quantitative PCR. Results were plotted relative to untreated levels. **(C) **NOTCH1 inhibition *in vivo *interferes with disease recurrence. Tumor-bearing MMTV-tTA/TOP-ICN1 transgenic mice were administered water containing doxycycline (10 μg/ml) for 28 days, and tumor volume was monitored externally by using calipers. Doxycycline was then removed from the water, and mice were monitored for tumor regrowth for an additional 40 days. Mice that failed to exhibit tumor regrowth were then killed and examined histologically for the presence of residual mammary tumor cells.

Previous studies suggest that human breast cancer cells become dependent on NOTCH1 in the absence of ERα or ERB2 signaling [15,18,20], raising the possibility that NOTCH inhibition may have therapeutic potential in TN human basal-like breast cancers. To determine whether NOTCH1 activity is required to maintain mammary tumor growth and survival *in vivo*, we administered doxycycline to tumor-bearing MMTV-tTA/TOP-ICN1 mice. Exposure to doxycycline to suppress intracellular NOTCH1 expression resulted in a 55% decrease in average tumor volume after 48 hours, and a >90% decrease in average tumor volume by day 9 (Figure 6A). To confirm that NOTCH1 signaling is impaired in regressing tumors, we isolated RNA from tumor-bearing mice left untreated or treated with doxycycline. Real-time quantitative PCR analysis revealed decreases in *Hes1, Deltex1*, and *c-Myc *expression levels in tumors isolated from dox-treated mice compared with untreated controls, thereby confirming repression of NOTCH1 signaling in the dox-treated mammary tumor-bearing mice (Figure 6B).

To determine whether NOTCH1 inhibition interferes with or prevents disease recurrence, we treated six tumor-bearing mice with doxycycline for 28 days, and then removed dox from the drinking water and monitored the animals for disease recurrence. Tumor regrowth was observed within 40 days of dox withdrawal in two of six tumor-bearing mice (Figure 6C). However, disease was not detected in the remaining four dox-treated mammary tumor-bearing mice, indicating that NOTCH1 inhibition was sufficient to prevent disease recurrence in these mice.

### Mammary tumor-initiating cells contribute to NOTCH1-mediated mammary tumorigenesis

Accumulating evidence suggests that certain tumors exhibit functional heterogeneity and that tumor initiation may be driven by a subset of cells designated tumor-initiating or tumor stem cells [53]. With a novel orthotopic mouse model, progress has been made in identifying a breast cancer stem or initiating population from TN breast cancer patients [54]. To determine whether NOTCH1-mediated mammary tumorigenesis is driven by a rare tumor-initiating cell, we performed an *in vivo *limiting-dilution assay. Four independent NOTCH1-driven mouse mammary tumors were injected as serial dilutions into the thoracic mammary fat pad of immunodeficient mice, and recipient mice were monitored for disease development. The frequency of mammary tumor-initiating cells was calculated by using the software program L-Calc (StemCell Technologies, Vancouver, Canada) and estimated to be 1/2,978 cells [95% confidence interval (95% CI), 1/5,689 to 1/1,559] (Table 1). This analysis revealed that the mammary tumor-initiating cell in this mammary tumor model is relatively rare and that the bulk of the NOTCH1-transformed mammary tumor cells lack the capacity to initiate disease in immunodeficient recipient mice.

**Table 1:** Limiting-dilution analysis of primary MMTV-tTA/TOP-ICN1 tumor cells

Number ofinjected cells	Number of micedeveloping tumors	Average latency(days)
10^6^	4/4	15.75 (± 3.5)
10^5^	7/7	20 (± 6.3)
10^4^	11/12	42 (± 13.6)
10^3^	6/14	43 (± 11.2)
10^2^	0/10	>100
Repopulating frequency (95% confidence interval), 1 in 2,978 (1/5,689 to 1/1,559).		

Number of positive outgrowths is shown as the number of mice developing tumors per the number of mice injected. Serial dilutions of primary tumor cells were resuspended in Matrigel, transplanted into the thoracic mammary fat pads of nude mice; transplanted mice were monitored for disease. Data are pooled from four independent mammary tumors. Tumor-repopulating frequency was calculated by using L-Calc limiting-dilution analysis software.

### NOTCH1 mediates tumorsphere colony activity *in vitro *{2nd level heading}

**Figure 7:**
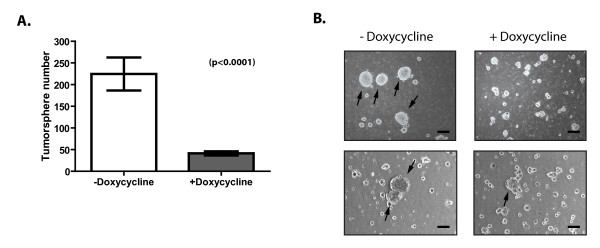
**NOTCH1 inhibition reduces mammary tumorsphere formation**. **(A) **Quantification of tumorsphere-forming potential of primary tumor cells isolated from dissociated MMTV-tTA/TOP-ICN1 tumors left untreated or treated with doxycycline (2 μg/ml) for 8 days. Error bars denote standard error (*n *= 3). **(B) **Representative images of untreated and doxycycline-treated mammary tumorspheres showing relative difference in tumorsphere size and morphology. Examples of spheres that were counted are indicated with arrows.

Our studies using doxycycline to suppress NOTCH1 activity in tumor-bearing mice suggest that NOTCH1 inhibition prevents or, at a minimum, delays disease recurrence (Figure 6C). We then tested whether NOTCH1 activity was required for mammary tumor-initiating cell activity by using an *in vitro *tumorsphere assay [38,55]. Tumorsphere-forming cells increase after neoadjuvant chemotherapy, and the molecular profile of tumors obtained after chemotherapy resembles the gene-expression profile of tumorsphere cells, suggesting that this assay enriches for tumor-initiating cells [56,57]. Primary NOTCH1-transformed mammary tumor cells formed spheres in culture at a rate of 1 in 268 (0.37%) (Figure 7A). Importantly, the ability of these cells to form tumorspheres *in vitro *remains dependent on the expression of intracellular NOTCH1, as treatment of primary mammary tumor cells with doxycycline results in a >75% decrease in the number of tumorspheres (Figure 7A, and see Additional file 5; *P *< 0.0001). The tumorspheres appeared enriched in Notch1-active cells, as *Deltex1 *expression was increased in the spheres compared with the primary tumors from which they were derived (Additional File 5). The tumorspheres that did emerge in the dox-treated cultures were noticeably smaller and less defined in structure than were the cultures in which intracellular NOTCH1 remained expressed (Figure 7B). Together, these data suggest that NOTCH1 contributes to mammary tumor-initiating activity *in vitro *and potentially *in vivo*.

## Discussion

Notch1 has been shown to promote commitment of mouse mammary stem cells along the luminal lineage [25,58]. Consistent with these and other studies [26,31-33], we show that constitutive expression of intracellular NOTCH1 in the developing mouse mammary gland stimulates luminal fate, ultimately resulting in transformation of the mammary gland. The mammary tumors predominantly express the luminal lineage marker keratin 8/18. Interestingly, in our model expression of human intracellular NOTCH1 in the developing mouse mammary gland did not result in induction of diverse tumor types that regressed upon weaning. Nor did the transgenic females exhibit any difficulty nursing their young. This is in contrast to transgenic models that constitutively express mouse ICN1 driven by the Mouse Mammary Tumor Virus (MMTV) LTR. These mice are unable to nurse their young and they develop lactation-dependent papillary tumors that regress upon involution [31,32]. The reasons for the phenotypic differences could reflect transgene expression levels or the timing of transgene induction since our model is doxycycline-regulated. Alternatively, human ICN1 may not interact with a mouse co-factor essential for lactation.

Using an *in vivo *limiting-dilution assay, we provide evidence that NOTCH1-transformed mammary tumors are functionally heterogeneous and estimate the frequency of mammary tumor-initiating cells to be approximately 1/3000 cells. We demonstrate that doxycycline treatment or NOTCH1 inhibition *in vivo *prevents disease recurrence in 4 of 6 mice examined. However, disease recurred within 21 days in 2 tumor bearing mice treated with dox, suggesting that NOTCH1 inhibition in these tumors was not sufficient to eliminate the tumor-initiating cells. These relapsed mammary tumors may contain increased numbers of mammary tumor-initiating cells and/or harbor genetic changes that render the tumors NOTCH1 independent.

Consistent with the *in vivo *limiting-dilution analyses, a subpopulation of NOTCH1 transformed mammary tumor cells grow in an *in vitro *tumorsphere assay and importantly, doxycycline treatment significantly reduces sphere number and size. The tumorsphere assays revealed that NOTCH1 is required both for the initiation and maintenance of tumorspheres *in vitro *and potentially for mammary tumor-initiating activity *in vivo*.

GSI treatment of ERB2-induced mouse mammary tumors reduced tumorspheres *in vitro *and interfered with the ability of the mammary tumor-initiating cells to induce disease in immunodeficient mice [59]. These studies are consistent with our findings and collectively suggest that NOTCH inhibitors may target mammary tumor-initiating cells driven by other oncogenes and not be limited to mammary tumors that exhibit NOTCH pathway activation.

NOTCH pathway activation has also been implicated in human mammary tumor-initiating cell biology. GSI treatment or treatment with an anti-NOTCH4 monoclonal antibody significantly reduces human tumorsphere formation *in vitro *[60]. Studies in the human breast cancer cell lines MCF7 and MDA-MB-231 show that NOTCH1 or NOTCH4 silencing reduces tumorsphere formation and inhibits tumor growth *in vivo*; however, NOTCH4 suppression appears to have the greatest inhibitory effect [61]. Thus, its possible that NOTCH4 is the relevant NOTCH receptor in human breast cancer-initiating cells.

To identify NOTCH1-regulated genes that might mediate mammary tumor-initiating cell activity, we applied transcriptional profiling to two mammary tumor cell lines in the absence/presence of doxycycline. We found the expression of several NOTCH1-regulated genes such as *Hes1, Hey1, Deltex1 *and *c-Myc *significantly reduced upon doxycycline treatment. In addition to these target genes, NOTCH1 activation stimulates expression of embryonic stem (ES) cell pluripotency transcription factor *Nanog*. The Nanog-Oct4-Sox2 (NOS) transcription factors activate self-renewal and inhibit differentiation in human and mouse ES cells and the NOS signature is enriched in claudin-low and basal-like breast cancer subtypes [62-64]. Consistent with these findings, we show that treatment of the ER-negative, basal-like human breast cancer cell line MDA-MB-231 with a gamma-secretase inhibitor reduces intracellular NOTCH1 and NANOG protein levels.

Like CD61, Nanog expression was not detected in the primary mouse mammary tumor tissue but was readily observed in the nuclei of the CD61-positive mammary tumor cell lines and tumorspheres. These data suggest that NOTCH1 regulation of Nanog may be cell-type or developmental stage specific. Thus, NOTCH1 may induce *Nanog *expression in luminal progenitors and mammary tumor-initiating cells but not in the bulk differentiated tumor cells. Although CSL sites are present in the mouse *Nanog *regulatory region, we were unable to demonstrate NOTCH1 or Mastermind-like 1 recruitment to the mouse *Nanog *locus, leading us to speculate that NOTCH1 may indirectly regulate *Nanog *expression in mammary tumor- initiating cells. Consistent with this hypothesis, ChIP-seq analysis has suggested that NOTCH1 binds the genome in association with the zinc finger protein ZNF143 [65] and Nanog expression in mouse ES cells has been linked to *Znf143 *regulation [66]. Thus, Notch1 and Znf143 may co-regulate *Nanog *expression in mammary tumor-initiating cells. Consistent with our findings in the mouse, siRNA studies have demonstrated that OCT4 and NANOG expression are required for human breast tumor-initiating activity [62].

## Conclusions

The relative resistance of breast cancer stem cells to conventional and targeted therapies highlights the need to develop agents able to target this population. Our findings in this NOTCH1 mammary tumor model implicate NOTCH1 as a potential therapeutic target in breast tumor-initiating cells.

## Abbreviations

CSL: CBF1/RBP-Jκ/Suppressor of Hairless/LAG-1; DCIS: ductal carcinoma *in situ*; EMT: epithelial mesenchymal transition; ER: estrogen receptor; ESC; embryonic stem cell; GSI: gamma secretase inhibitor; HER-2: human epidermal growth factor receptor-2; ICN1: human intracellular NOTCH1; MaSC,: mammary stem cell; NOS: Nanog-Oct4-Sox2; PR: progesterone receptor; TN: triple negative.

## Competing interests

The authors declare that they have no competing interests.

## Authors' contributions

MS carried out all the experiments with assistance from NH. RS assisted with the chromatin immunoprecipitation experiments. MS and MK designed the experiments, analyzed the data, and wrote the manuscript. All authors read and approved the manuscript.

## Authors' details

^1^MS, NH, and MK are members of the Department of Cancer Biology at the University of Massachusetts Medical School.

^2^RS is a member of the Program in Gene Function and Expression, University of Massachusetts Medical School. Worcester, MA, USA.

## Supplementary Material

Additional file 1 **Nulliparous MMTV-tTa/TOP-ICN1 mice express increased levels of intracellular NOTCH1 compared with littermate controls**. Age-matched, nulliparous MMTV-tTa/TOP-ICN1 mice and littermate controls were left untreated or were administered doxycycline (10 μg/ml) in their drinking water for 7 days. Mammary glands were isolated, and intracellular NOTCH1 protein levels were determined with immunoblotting. ERK1/2 was used as a loading control. Click here for file

Additional file 2 **NOTCH1 activity does not significantly alter CD61 expression levels**. Mammary tumor cell lines were left untreated or were treated with doxycycline (2 μg/ml) for 24 hours. Total RNA was harvested and *CD61, Hes1*, and *Deltex1 *mRNA levels determined using quantitative real-time PCR. The figure represents an average of two independent cell lines. Click here for file

Additional file 3 **Nulliparous MMTV-tTA/TOP-ICN1 transgenic mice develop mammary tumors after a long latency**. A cohort of nulliparous MMTV-tTA/TOP-ICN1 mice (*n *= 14) and littermate controls (*n *= 15) were monitored for tumor formation and compared with cohorts of MMTV-tTA/TOP-ICN1 transgenic mice (*n *= 65) maintained under mating conditions. The proportion of tumor-free mice was plotted by using the Kaplan-Meier software. Click here for file

Additional file 4 **Mammary tumor-derived cell lines produce tumors morphologically identical to primary tumors**. Tumor-derived cell lines were injected into the mammary fat pads of nude mice, and the resulting tumors were fixed in 10% formalin, paraffin embedded, sectioned and stained with hematoxylin and eosin (H&E). Representative fields are shown at 400X magnification. Click here for file

Additional file 5 **Mammary tumorspheres are enriched in NOTCH1 activity and remain doxycycline responsive**. Primary mammary tumor cells were plated in the tumorsphere assay and were left untreated or were treated with 2 μg/ml doxycycline for 24 hours. Total RNA was harvested from pooled spheres, and *Deltex1 *mRNA levels were determined using quantitative real-time PCR. The figure represents the average from two independent experiments and was normalized to primary tumor RNA. Click here for file
