# Supplementary figures and images for: NOTCH1 inhibition in vivo results in mammary tumor regression and reduced mammary tumorsphere-forming activity in vitro
Source: Breast Cancer Res. 2012 Sep 19;14(5):R126. doi: 10.1186/bcr3321 (PMC4053103; doi:10.1186/bcr3321)

# Additional File 1

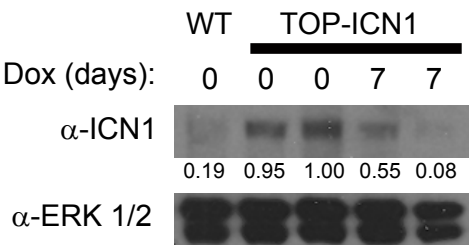

Supplement: Additional file 1 — Nulliparous MMTV-tTa/TOP-ICN1 mice express increased levels of intracellular NOTCH1 compared with littermate controls. Age-matched, nulliparous MMTV-tTa/TOP-ICN1 mice and littermate controls were left untreated or were administered doxycycline (10 μg/ml) in their drinking water for 7 days. Mammary glands were isolated, and intracellular NOTCH1 protein levels were determined with immunoblotting. ERK1/2 was used as a loading control. [file bcr3321-S1.PDF]

# Additional File 2

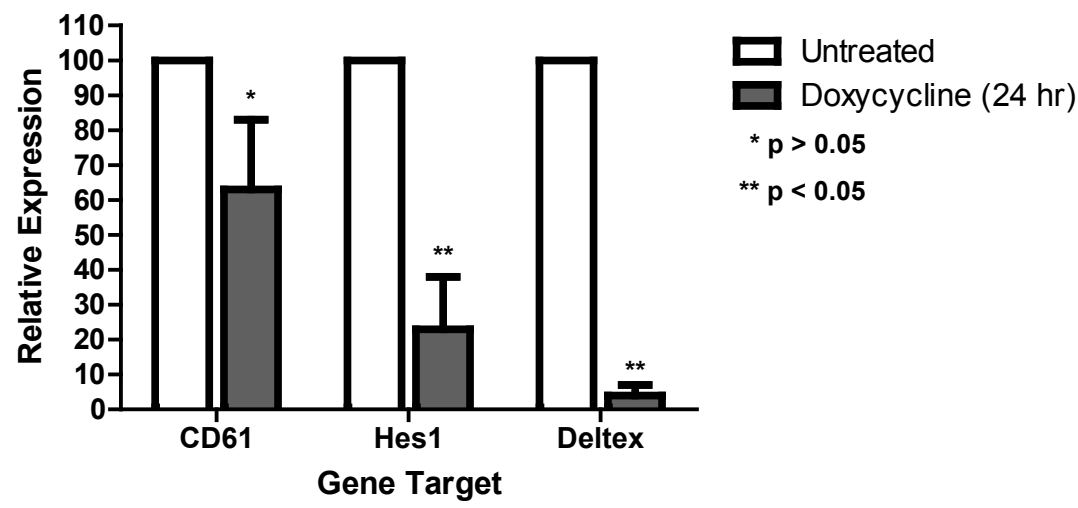

Supplement: Additional file 2 — NOTCH1 activity does not significantly alter CD61 expression levels. Mammary tumor cell lines were left untreated or were treated with doxycycline (2 μg/ml) for 24 hours. Total RNA was harvested and CD61, Hes1, and Deltex1 mRNA levels determined using quantitative real-time PCR. The figure represents an average of two independent cell lines. [file bcr3321-S2.PDF]

# Additional File 3

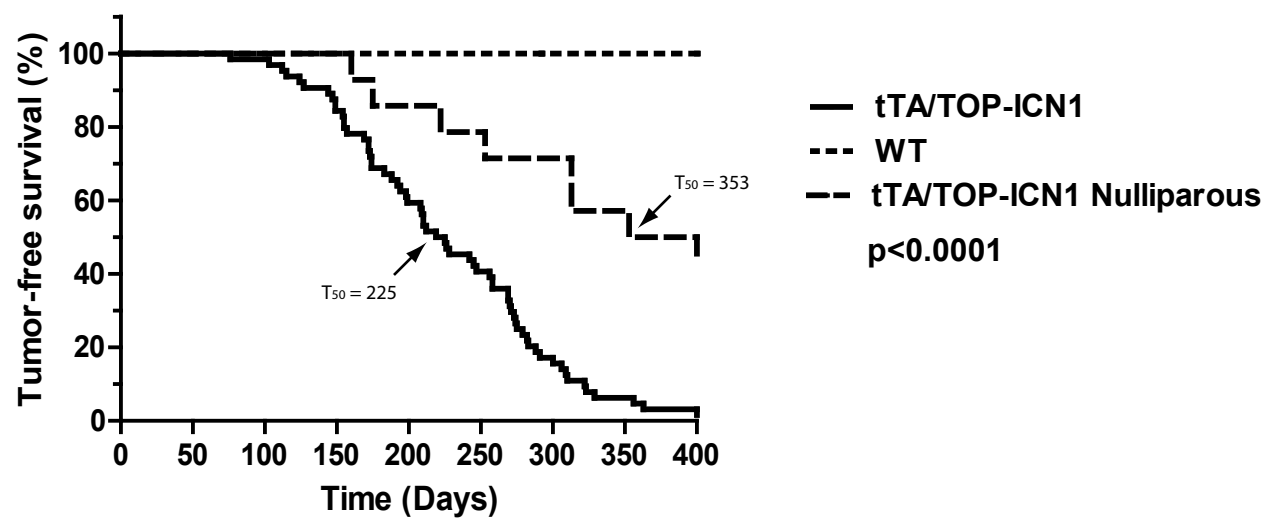

Supplement: Additional file 3 — Nulliparous MMTV-tTA/TOP-ICN1 transgenic mice develop mammary tumors after a long latency. A cohort of nulliparous MMTV-tTA/TOP-ICN1 mice (n = 14) and littermate controls (n = 15) were monitored for tumor formation and compared with cohorts of MMTV-tTA/TOP-ICN1 transgenic mice (n = 65) maintained under mating conditions. The proportion of tumor-free mice was plotted by using the Kaplan-Meier software. [file bcr3321-S3.PDF]

# Additional File 4

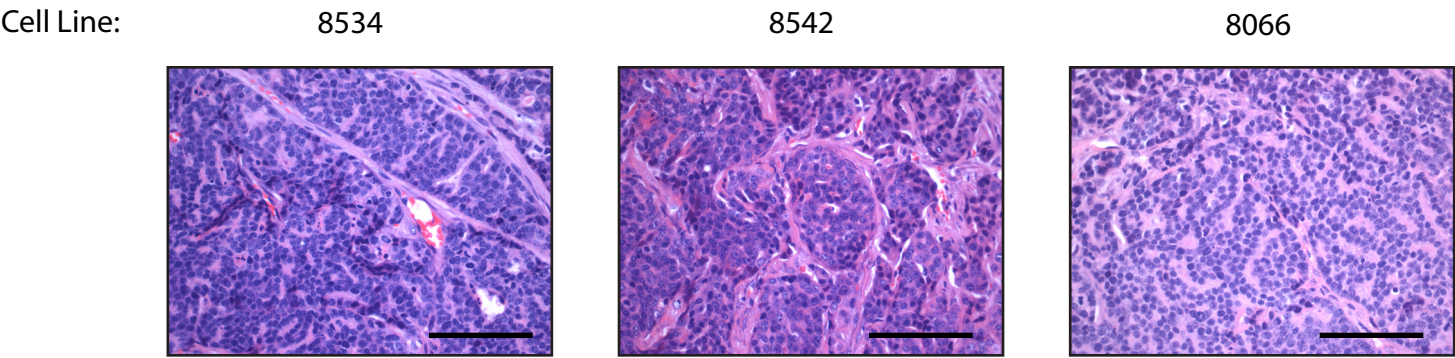

Supplement: Additional file 4 — Mammary tumor-derived cell lines produce tumors morphologically identical to primary tumors. Tumor-derived cell lines were injected into the mammary fat pads of nude mice, and the resulting tumors were fixed in 10% formalin, paraffin embedded, sectioned and stained with hematoxylin and eosin (H&E). Representative fields are shown at 400X magnification. [file bcr3321-S4.PDF]

Additional File 5

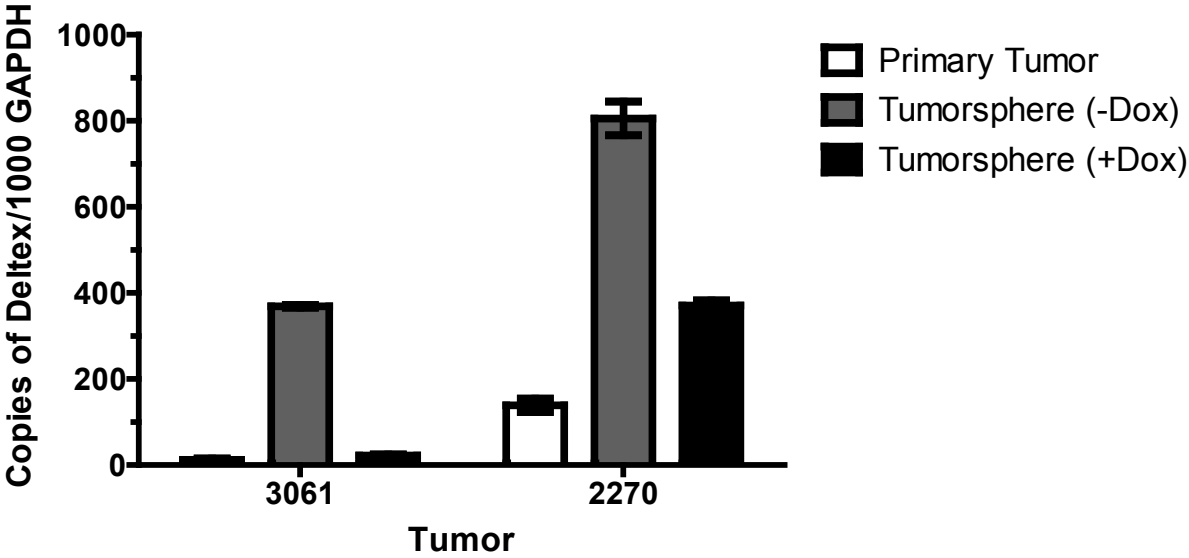

Supplement: Additional file 5 — Mammary tumorspheres are enriched in NOTCH1 activity and remain doxycycline responsive. Primary mammary tumor cells were plated in the tumorsphere assay and were left untreated or were treated with 2 μg/ml doxycycline for 24 hours. Total RNA was harvested from pooled spheres, and Deltex1 mRNA levels were determined using quantitative real-time PCR. The figure represents the average from two independent experiments and was normalized to primary tumor RNA. [file bcr3321-S5.PDF]
